# Embosphere microspheres size for bronchial artery embolization in patients with hemoptysis caused by bronchiectasis: a retrospective comparative analysis of 500–750 versus 700–900 μm microspheres

**DOI:** 10.1186/s12890-024-03019-4

**Published:** 2024-04-24

**Authors:** Hong-Dou Xu, Liang Yang, Shi-Bing Hu

**Affiliations:** Department of Interventional Radiology, Gaochun Peoples Hospital Affiliated to Jiangsu University, 53 Maoshan Road, Gaochun District, Nanjing, 211302 Jiangsu China

**Keywords:** Size, Bronchiectasis, Embosphere microspheres, Hemoptysis, Hemoptysis recurrence

## Abstract

**Background:**

Bronchial arterial embolization (BAE) has been accepted as an effective treatment for bronchiectasis-related hemoptysis. However, rare clinical trials compare different sizes of specific embolic agents. This study aims to evaluate whether different Embosphere microsphere sizes change the outcome of BAE.

**Methods:**

A retrospective review was conducted on consecutive patients with bronchiectatic hemoptysis who were scheduled to undergo BAE treatment during a period from January 2018 to December 2022. The patients received BAE using microspheres of different sizes: group A patients were treated with 500–750 μm microspheres, and group B patients were treated with 700–900 μm microspheres. The cost of embolic microspheres (Chinese Yuan, CNY), duration of hospitalization, complications, and hemoptysis-free survival were compared between patients in group A and those in group B. A Cox proportional hazards regression model was used to identify predictors of recurrent hemoptysis.

**Results:**

Median follow-up was 30.2 months (range, 20.3–56.5 months). The final analysis included a total of 112 patients (49–77 years of age; 45 men). The patients were divided into two groups: group A (*N* = 68), which received 500–750 μm Embosphere microspheres, and group B (*N* = 44), which received 700–900 μm Embosphere microspheres. Except for the cost of embolic microspheres(group A,5314.8 + 1301.5 CNY; group B, 3644.5 + 1192.3 CNY; *p* = 0.042), there were no statistically significant differences in duration of hospitalization (group A,7.2 + 1.4 days; group B, 8 + 2.4days; *p* = 0.550), hemoptysis-free survival (group A, 1-year, 2-year, 3-year, 85.9%, 75.8%, 62.9%; group B, 1-year, 2-year, 3-year, 88.4%, 81.2%,59.4%;*P* = 0.060), and complications(group A,26.5%; group B, 38.6%; *p* = 0.175) between the two groups. No major complications were observed. The multivariate analysis results revealed that the presence of cystic bronchiectasis (OR 1.61, 95% CI 1.12–2.83; *P* = 0.001) and systemic arterial-pulmonary shunts (SPSs) (OR 1.52, 95% CI 1.10–2.72; *P* = 0.028) were independent risk factors for recurrent bleeding.

**Conclusions:**

For the treatment of BAE in patients with bronchiectasis-related hemoptysis, 500–750 μm diameter Embosphere microspheres have a similar efficacy and safety profile compared to 700–900 μm diameter Embosphere microspheres, especially for those without SPSs or cystic bronchiectasis. Furthermore, the utilization of large-sized (700–900 μm) Embosphere microspheres is associated with the reduced cost of an embolic agent.

**Supplementary Information:**

The online version contains supplementary material available at 10.1186/s12890-024-03019-4.

## Background

As a chronic respiratory disease, bronchiectasis is characterized by irreversible bronchial dilatation [1]. Patients with bronchiectasis often experience chronic respiratory symptoms, such as cough, purulent sputum, hemoptysis, dyspnea, and recurrent infections. Moreover, approximately 70% of Chinese bronchiectasis patients experience hemoptysis, placing a burden on healthcare systems [2]. For these patients, many studies have shown that bronchial artery embolization (BAE) is an effective and minimally invasive option [3–10]. However, the rate of clinical failure after PAE is relatively high. Specifically, 5% of patients still have moderate to severe hemoptysis within 1 month of embolization, and long-term results show that approximately 20% of patients have a recurrence of hemoptysis after successful embolization [4].

In BAE, the culprit bronchial artery is cannulated and typically embolized using a variety of embolizers, including coils, polyvinyl alcohol (PVA), microspheres, gelatin sponges, etc. The efficacy of PVA and microspheres as embolic agents for BAE has been established through clinical experience and experimental studies, making them the prevailing standard [3–10, 16–19]. In comparison to PVA, microspheres exhibit several benefits, including precise sizing, resistance to aggregation, and satisfactory elasticity [6, 7]. Despite their excellent physical properties, microspheres for the treatment of bronchial artery embolization (BAE) in patients with hemoptysis have shown similar clinical results to PVA, according to a recent study [9]. Nonetheless, many studies compare different embolic agents, and we have found only a few clinical trials that compare different sizes of specific embolic agents. Furthermore, the determination of the optimal size of Embosphere microspheres for BAE remains inconclusive, as the majority of centers employ particles with diameters ranging from 500 to 750 μm or 700–900 μm [2–8].

The purpose of this retrospective study was to compare the efficacy and safety profiles of microspheres 500–750 versus (vs.) 700–900 μm in size for BAE treatment in patients with bronchiectasis-related hemoptysis, which helped to provide evidence for embolic agent selection.

## Methods

### Patients

**Fig. 1:**
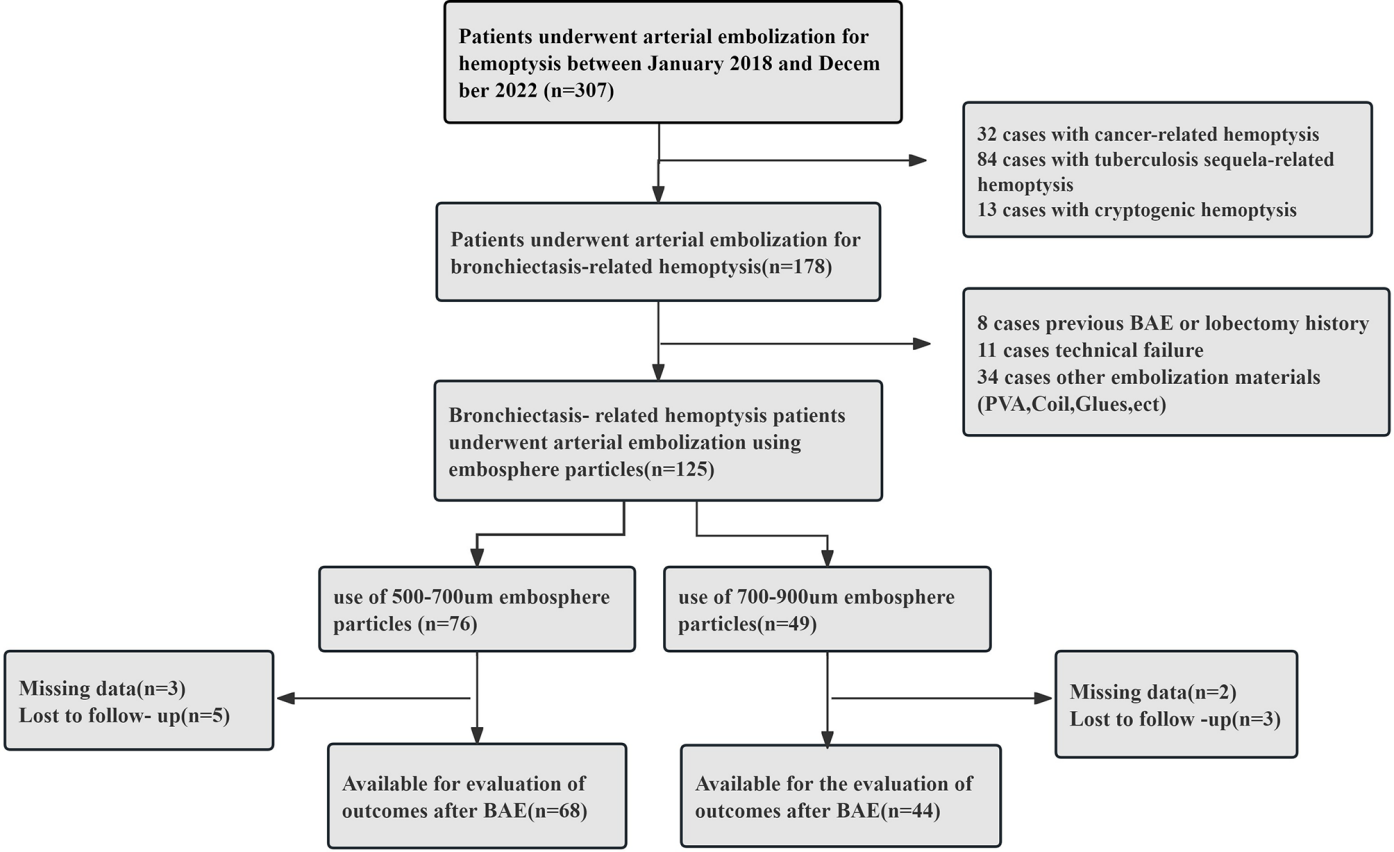
Flowchart of enrolled patients

From January 2018 to December 2022, a total of 307 consecutive patients who underwent arterial embolization treatment for bronchiectasis-related hemoptysis in our hospital were included in the study. The inclusion criteria were (1) confirmed hemoptysis (a volume of hemoptysis of more than 20 ml in each event and ineffectiveness of standard medical therapy); (2) diagnosed with bronchiectasis based on computed tomography (CT) scan with a ratio of the cross-sectional diameter of the inner airway to its accompanying artery > 1.0; and (3) about to receive BAE treatment with the use of Embosphere microspheres. The exclusion criteria were (1) a history of lobectomy or BAE for hemoptysis.; (2) missing clinical information or loss to follow-up; and (3) technical failure. In total, 112 patients with bronchiectasis-related hemoptysis were enrolled for analysis. A flowchart of the enrolled patients is shown in Fig. 1. Patients were classified into two groups based on the diameter of Embosphere microspheres, namely, 500–750 μm (Group A, *N* = 68) and 700–900 μm (Group B, *N* = 44). All patients were evaluated with contrast-enhanced computed tomography to identify the culprit vessels of hemoptysis. This study was approved by the Ethics Committee of our hospital, and the requirement for informed consent was waived because of the retrospective nature of the study.

### Bronchial artery embolization procedures

The procedures were conducted under local anesthesia with 2% lidocaine hydrochloride. The vital signs of each patient were monitored throughout the procedure. A 5-F vascular sheath was placed in the common femoral artery. Under the guidance of DSA, angiographic catheters (Simon catheter, left gastric catheter, or Cobra catheter, Cordis, USA) were selectively catheterized into the offending vessels. A swan-neck microcatheter 2.4–2.8 F (Merit Maestro, USA) was introduced coaxially into the angiographic catheter and advanced through the pathologic arteries to a possible distal point near the blush. Bronchial angiograms were performed. These culprit arteries were embolized with microspheres 500–750 μm and 700–900 μm (Embosphere Microspheres; Merit Medical, USA) as the embolic agent.

### Study endpoints and follow-up

The study endpoints included recurrent hemoptysis and in-hospital clinical outcomes. The length of hospital stay was defined as the number of days from the presentation of hemoptysis for patients hospitalized to discharge. In-hospital clinical outcome was evaluated by the cost of the embolic microsphere, complications, and length of hospital stay. The cost of an embolic microsphere is defined as the bottles used multiplied by unit prices. Complications associated with the procedure that led to extended hospitalization, escalated medical attention, enduring sequelae, or mortality were classified as significant complications according to the Society of Interventional Radiology’s guidelines [10]. Two thoracic radiologists independently evaluated the characteristics of non-contrast CT images, and any discrepancies were resolved through consensus with a third radiologist. The type of bronchiectasis was described as columnar, varicose, or cystic according to the Reid classification [11]. The severity of bronchial dilatation was a modified version of that described by Reiff et al. [12] (0 = normal, 1 = less than twice the diameter of the accompanying pulmonary artery, 2 = 2–3 times the diameter of the accompanying pulmonary artery, 3 = more than 3 times the diameter of the accompanying pulmonary artery).

Regular follow-up by telephone or clinical visits was performed after hospital discharge. During the follow-up period, the status of hemoptysis and any adverse events were recorded. Recurrence was defined as hemoptysis volume ≥ 30 mL/d, need for repeat BAE, need for lobectomy, or death due to recurrence. Recurrence-free time was defined as the interval between the date of hemostasis during hospitalization and either the date of recurrence or the date of the last follow-up (May 2023, for patients with available data).

### Statistical analysis

Continuous variables are presented as the mean ± standard deviation or median with an interquartile range. Categorical variables were compared between groups using the χ2 test or Fisher’s exact test, and continuous variables were compared using the t-test or Wilcoxon test. Recurrence-free survival was estimated by the Kaplan‒Meier method, and the log-rank test was used to identify differences between recurrence-free survival curves. Patients were censored at the time of death if they died of causes other than hemoptysis. Univariate and multivariate Cox proportional hazards regression models were used to identify predictive factors for recurrent hemoptysis. Regression models and factors with a P value < 0.05 in the univariable analysis were included in the multivariable analysis using the enter method. Data were analyzed by R language (Version 3.5.3), and *P* < 0.05 was considered statistically significant.

## Results

### Clinical characteristics of patients with hemoptysis

There were 68 patients in group A and 44 patients in group B. The mean age in group A was 60.2 ± 11.6 years, with 41 (41/68, 60.3%) females and 27 (27/68, 39.7%) males. For group B, the mean age was 49.4 ± 13.7 years, and the numbers of females and males were 26 (26/44, 59.1%) and 18 (18/44, 40.9%), respectively. The baseline characteristics are shown in Table 1. The variables did not demonstrate statistically significant differences (*p* > 0.05). The bronchial angiographic findings of the patients are summarized in Table 2. No statistically significant differences were observed between the two groups.

**Table 1:** Clinical features of the study population(*N* = 112)

Parameters	Group A(*n* = 68)	Group B(*n* = 44)	P value
Age (years), mean ± SD	60 ± 11	49 ± 13	0.217
Female/Male, No. (%)	41(60.3)/27(39.7)	26(59.1)/18(40.9)	0.899
Volume of hemoptysis(ml/d), No. (%)			0.460
< 100 ml	32(47.0)	15(34.1)	
100—300 ml	25(36.8)	14(31.8)	
> 300 ml	18(26.4)	15(34.1)	
History of smoking, No. (%)	27(39.7)	23(52.3)	0.191
Comorbidities, No. (%)			0.679
Hypertension	9(13.2)	7(15.9)	
Hepatitis	3(4.4)	1(2.2)	
Cerebral infarction	2(2.9)	3(6.8)	
Diabetes mellitus	4(5.9)	4(9.1)	
The number of lung segments involved	3(1–5)	6(2–8)	0.151
Bronchial dilatation severity score	1.9(0.8–2.5)	2.1(1.8–2.5)	0.072
Bronchiectasis type, No. (%)			0.112
Columnar	11(16.2)	6(13.6)	
Varicose	39(57.4)	18(40.9)	
Cystic	18(26.5)	20(45.6)	

Note: Group A is presented as Embosphere microspheres of 500–750 μm, and Group B is presented as Embosphere microspheres of 700–900 μm. Continuous data are presented as the standard deviation or median (interquartile range); SPSs systemic arterial-pulmonary circulation shunts; *p < 0.05 indicates statistical significance.

**Table 2:** Details of the procedure

Parameter	GroupA(*n* = 68)	GroupB(*n* = 44)	p-value
Angiographic findings, No. (%)			0.311
Hypervascularity	59(86.8)	36(81.8)	
Hypertrophy	32(47.1)	23(52.3)	
SPSs	34(50.0)	13(29.5)	
The average number of culprint vessels	2.65	3.00	0.350
Types of culprit vessels, No. (%)			0.997
Bilateral BA	33(48.5)	23(52.3)	
Bilateral BA + others♦	16(23.5)	11(25)	
Right BA	9(13.2)	6(13.6)	
Right BA + others♦	4(5.9)	2(4.5)	
Left BA	3(4.4)	1(2.3)	
Left BA + others♦	3(4.4)	1(2.3)	

Note: Group A is presented as Embosphere microspheres of 500–750 μm, Group B is presented as Embosphere microspheres of 700–900 μm, and continuous data are presented as the mean ± standard deviation or median (interquartile range)

### Comparison of hemoptysis recurrence rate

**Fig. 2:**
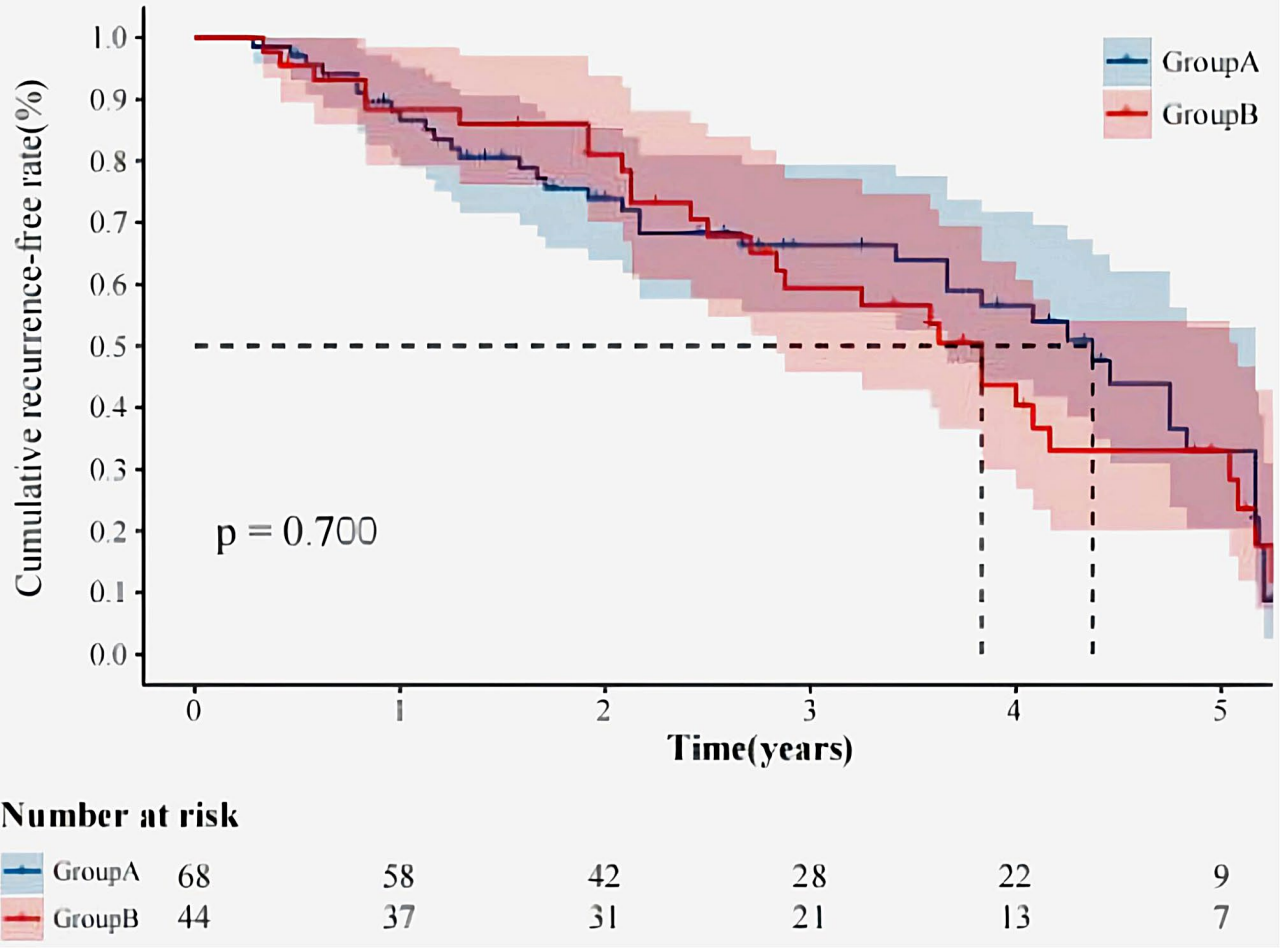
Comparison of the estimated cumulative recurrence-free rate curves for patients between Group A (500–750 μm Embosphere microsphere particles) and Group B (700–900 μm Embosphere microspheres). The parentheses indicate the number of patients at risk

Median follow-up was 30.2 months (range, 20.3–56.5 months). During follow-up, recurrence of hemoptysis occurred in a total of 69 (69/122, 61.6%) patients (45 in group A, 24 in group B). The hemoptysis status was recorded and analyzed, and no statistically significant differences were observed between the two groups (*P* = 0.06). The 1-year, 2-year, and 3-year hemoptysis-free survival rates were 85.9%, 75.8%, and 62.9% for group A and 88.4%, 81.2% and 59.4% for group B, respectively (Fig. 2).

**Fig. 3:**
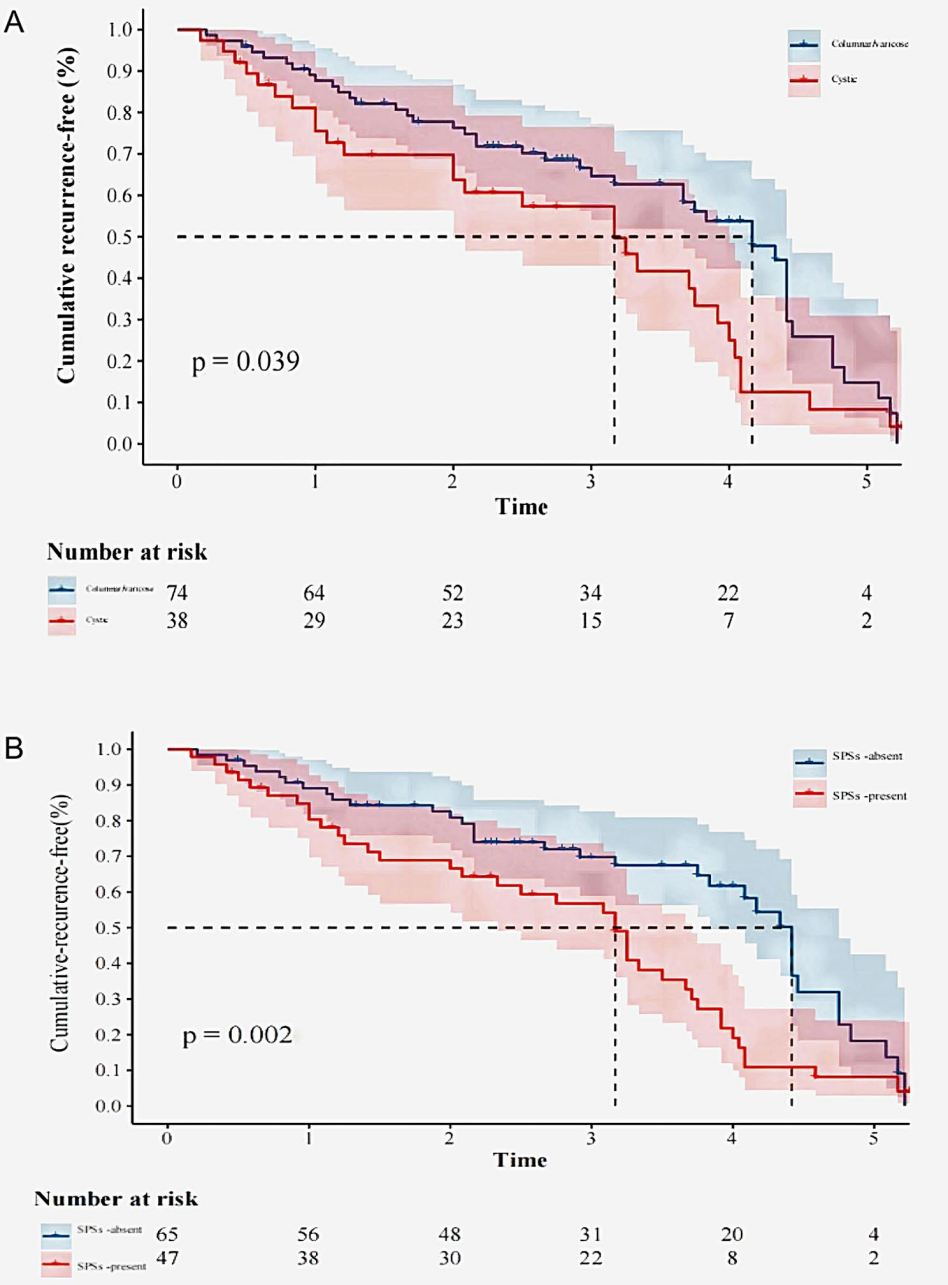
Comparison of the estimated cumulative recurrence-free rate curves for patients according to predictors of recurrent hemoptysis. Recurrence-free rate curves for the patients with or without cystic bronchiectasis. **B** Recurrence-free rate curves for the patients with or without SPSs. SPSs systemic arterial-pulmonary circulation shunts

Univariate and multivariate analyses of variables associated with recurrence are shown in Table 3. Multivariate analysis revealed that SPSs [hazard ratio (HR), 1.61; 95% confidence interval (CI), 1.12–2.83; *P* < 0.001] and the bronchiectasis subtype (the level of cystic) (HR, 1.52; 95% CI, 1.12–2.84; *P* = 0.028) were independently associated with recurrence. The Kaplan–Meier estimated curves of recurrence-free survival by multivariate analysis for patients with or without cystic bronchiectasis and SPSs are shown in Fig. 3.

**Table 3:** Univariate and multivariate analyses of the variables associated with the recurrence of hemoptysis in the patients after BAE treatment

Parameters	Recurrence (*n* = 69)	Nonrecurrence (*n* = 43)	Univariate		Multivariate	
			HR (95% CI)	P value	HR (95% CI)	P value
Age(years), mean ± SD	58 ± 11	57 ± 13	0.98(0.95–1.01)	0.322		
Sex						
Female, No. (%)	43(62.3)	24(55.8)	1	-		
Male, No. (%)	26(37.7)	19(44.2)	0.67(0.52–1.39)	0.095		
Volume of hemoptysis (ml/d), No. (%)						
< 100 ml	34(49.3)	13(30.2)	1.21(0.50–2.12)	0.660		
100–300 ml	23(33.3)	16(37.2)	1.37 (0.35–6.45)	0.530		
> 300 ml	12(17.4)	14(32.6)	2.45 (1.28–6.85)	0.018*	0.98(0.89–1.05)	0.389
History of smoking, No. (%)	24(34.8)	26(60.5)	1.32 (0.74–7.28)	0.435		
Comorbidities, No. (%)						
Hypertension	10(14.5)	6(14.0)	1.05 (0.65–1.85)	0.998		
Hepatitis	2(2.9)	2(4.7)	1.00 (0.98–1.02)	0.895		
Cerebral infarction	3(4.3)	2(4.7)	1.03 (0.38–3.54)	0.978		
Diabetes mellitus	5(7.2)	3(7.0)	1.00 (0.99–1.20)	0.435		
The number of lung segments involved	5(2,8)	4(2,7)	1.04 (0.99–1.13)	0.124		
Bronchial dilatation severity score	2.0(1.6,2.6)	1.7(1.1,2.5)	1.15 (0.85–1.66)	0.125		
Bronchiectasis type, No. (%)						
Columnar/varicose	34(49.3)	40(93.0)	1	-		
Cystic	32(46.4)	6(14.0)	2.52 (1.55–5.07)	< 0.001*	1.52(1.10–6.72)	0.028*
The diameter of embosphere microspheres, No. (%)						
500–750 μm	45(65.2)	24(55.8)	0.52 (0.15–1.38)	0.170		
700–900 μm	24(34.8)	19(44.2)	0.61 (0.52–1.98)	0.410		
Presence of SPSs, No. (%)	37(53.6)	10(23.3)	2.47(1.62–6.31)	<0.001*	1.61(1.12–5.83)	< 0.001*
The average number of cul print vessels	3.25	2.65	2.27(0.52–3.89)	0.462		
Presence of culprit NBSAs, No. (%)	22(31.9)	15(34.9)	1.81(0.75–4.31)	0.183		

Note: Continuous data are presented as the mean standard deviation or median (interquartile range). HR hazard ratio, CI confidence interval, BAE bronchial artery embolization, SPSs systemic arterial-pulmonary circulation shunts, NBSAs nonbronchial systemic arteries, *p < 0.05 indicates statistical significance

### Comparison of in-hospital clinical outcomes

**Fig. 4:**
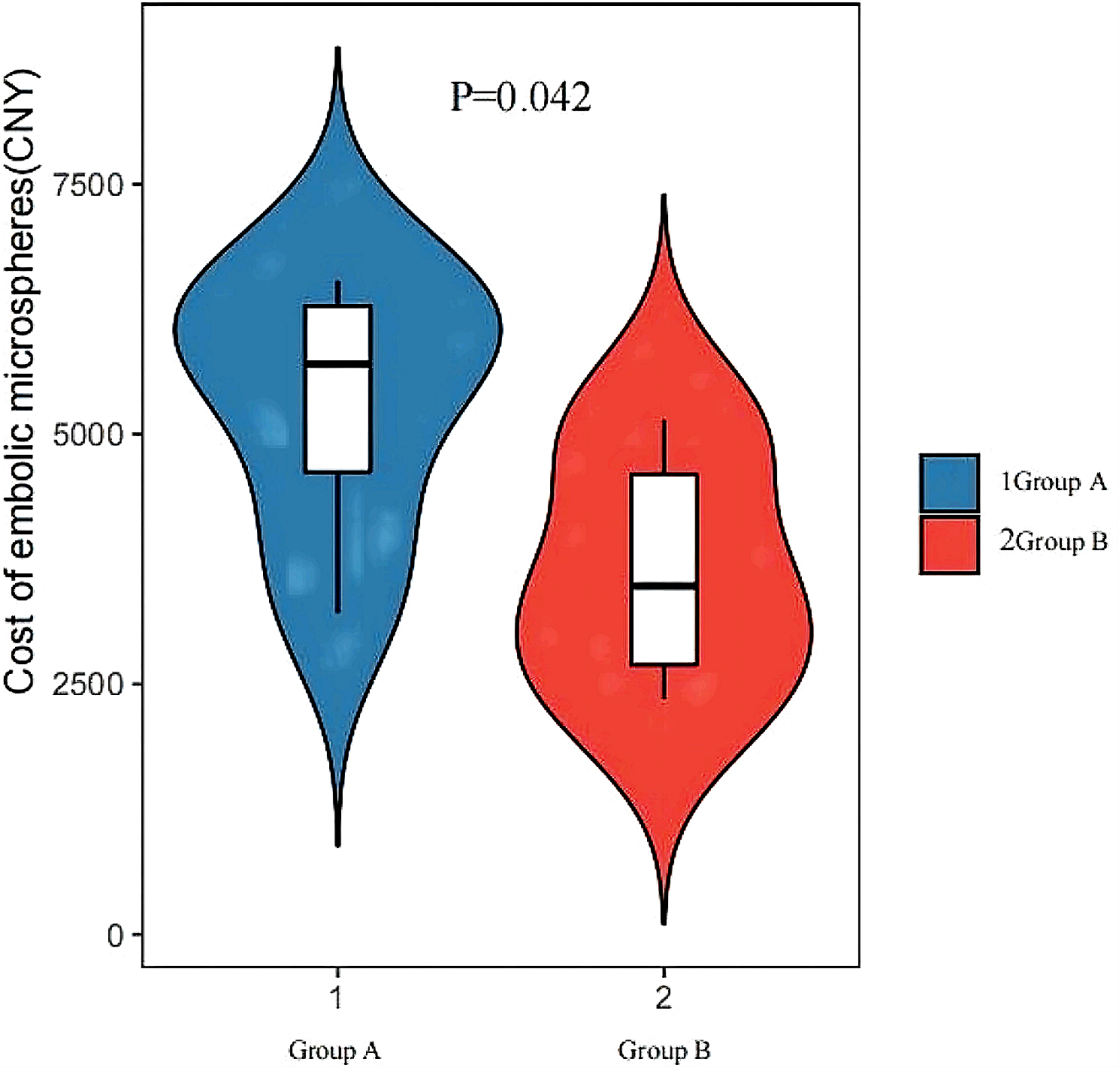
Comparison of the cost of embolic microspheres between Group A (500–750 μm Embosphere microspheres) and Group B (700–900 μm Embosphere microspheres)

The in-hospital clinical outcomes are summarized in Table 4. No statistically significant differences were observed between the two groups except for the cost of embolic microspheres (group A, 5314.8 ± 1301.5 CNY; group B, 3644.5 ± 1192.3 CNY; *p* = 0.042) (Fig. 4). The length of hospital stay was 7.2 ± 1.48 days in group A and 8 ± 2.45 days in group B (*p* = 0.550). No major complications were observed. The complication rate was 18 (26.5%) in group A and 17 (38.6%) in group B (*p* = 0.175). In detail, there was no difference in cough/expectoration (*P* = 1.000), nausea/vomiting (*P* = 1.000), abdominal/chest pain (*P* = 0.430), ecchymosis/hematoma at the puncture site (*P* = 0.393), allergy and dyspnea (*P* = 1.000), or fever (*P* = 0.212) between the two groups.

**Table 4:** In-hospital clinical outcomes in all patients after BAE treatment (*N* = 112)

Parameter	Group A(*n* = 68)	Group B(*n* = 44)	P value
length of hospital stay, days	7.2 + 1.4	8 + 2.4	0.550
Cost of embolic microspheres(CNY)	5314.8 + 1301.5	3644.5 + 1192.3	0.042*
Complication, No. (%)	18(26.5)	17(38.6)	0.175
Major	0(%)	0(%)	-
Mnior	18(26.5)	17(38.6)	0.175
Cough/expectoration	1(1.5)	0(0)	1.000
Nausea/vomiting	7(10.3)	4(9.1)	1.000
Abdominal/chest pain	3(4.4)	4(9.1)	0.430
Ecchymosis/hematoma at the puncture site	0(0)	1(2.3)	0.393
Allergy and dyspnea	2(2.9)	1(2.3)	1.000
Fever	5(7.4)	7(15.9)	0.212

Note: Group A is presented as Embosphere microsphere particles of 500–750 μm, and Group B is presented as Embosphere microsphere particles of 700–900 μm. Continuous data are presented as the mean ± standard deviation or median (interquartile range). CNY China yuan, *p < 0.05 indicates statistical significance

## Conclusions

In this retrospective study, we evaluated the treatment effectiveness and safety profile of BAE with smaller (500–750 μm) versus larger (700–900 μm) Embosphere microspheres for the treatment of bronchiectasis-related hemoptysis. We found that larger microspheric particles (700–900 μm in size) have a significantly lower cost of embolic microspheres. However, the duration of hospitalization, complications, and hemoptysis-free survival rates (1 year, 2 years, and 3 years) were similar between groups. Furthermore, our results indicated that the cystic type of bronchiectasis and SPSs were independent predictive factors for increased hemoptysis recurrence risk.

In recent decades, various agents have been employed for bronchial artery embolization. Nonabsorbable Embosphere microspheres, which are relatively new embolic agents, have several advantages, including excellent biocompatibility, aggregation resistance, and satisfying elasticity [9, 13]. As a result, they benefit from preventing microcatheter occlusion and have been widely used to treat various diseases. For example, a prospective multicenter clinical trial in Japan illustrated that transarterial embolization (TAE) using tris acryl gelatin microspheres (TGMs) for hypervascular tumors was technically feasible and safe for devascularization [14]. In addition, a recent study illustrated that embosphere embolic microspheres have good efficacy and safety in the treatment of prostatic hyperplasia rupture and hemorrhage, with a mild adverse reaction compared to the gel foam embolic agent [15]. On the other hand, the efficacy and safety of BAE with PVA in the control of hemoptysis was demonstrated. PVA is also nonabsorbable but has a nonuniform shape, resulting in an increased tendency for clumping, which may cause catheter or proximal arterial obstruction during infusion, which may increase the risk of recurrent hemoptysis in the long term [6, 7, 16, 17]. However, a previous study demonstrated similar efficacy and safety profiles of microspheres versus PVA for BAE in patients with hemoptysis [9]. To the best of our knowledge, the different sizes of specific embolic agents for BAE for patients with hemoptysis due to varying causes have not been properly investigated to date.

In our study, we showed that the hemoptysis-free survival rates were (group A vs. group B: 85.9% vs. 88.4%) at 1 year, (group A vs. group B: 75.8% vs. 81.2%) at 2 years, and (group A vs. group B: 62.9% vs. 59.4%) at 3 years. Even though we should interpret these data with caution since patient prognoses may differ depending on their underlying diseases, the procedural outcomes were favorable and comparable with those of other embolization materials, such as metallic coils, PVA, or NBCA. For example, Ishikawa et al. [18] reported that the 1-year and 3-year hemoptysis-free survival rates of BAE with metallic coils (*n* = 489) were 86.9% and 57.6%, respectively. Woo et al [19] reported that the 1-year and 3-year hemoptysis-free survival rates of ssBAE with PVA (*n* = 293) were 77% and 68%, respectively, and those with NBCA (*n* = 113) were 88% and 85%, respectively. However, the incidence of recurrent hemoptysis in our study was slightly higher than that reported in the data using 300–500 μm Embosphere microspheres (*n* = 90), which reported that the 1-year hemoptysis-free survival rate after BAE was 91.1% [9]. The possible explanations might include the following: (1) The sample size in our study was relatively large, which could provide more statistical validation. (2) The higher proportion of patients with cystic bronchiectasis, has been associated with a very high likelihood of recurrent bleeding. Moreover, based on the evidence to date, the particle size selected for a particular embolization procedure is determined by the desired level of occlusion (i.e., proximal or distal). In general, the use of small particles results in a more distal occlusion. Microparticles with larger diameters might have a disadvantage in occluding the index bronchial artery more proximally than preferred, which could lead to recurrent hemoptysis from systemic collaterals [20]. However, we did not observe a decreased hemoptysis recurrence rate in Embosphere microspheres of 500–750 μm compared with 700–900 μm microspheres in our study. The results were similar to those of a previous study. Although Kucukay [6] used Embosphere microspheres with a diameter of 700–900 μm and Corr [7] used microspheres with a diameter of 500–750 μm, there was no significant difference in the rate of recurrence of hemoptysis (8.1 vs. 13%). The possible reasons include the following: (1) This risk of hemoptysis recurrence can be diminished by using a microcatheter. In other words, placing it in a possible closer location near the abnormal vasculature results in less recurrent hemoptysis from nonbronchial systemic collaterals. (2) Based on the available evidence, the main causes of recurrent hemoptysis were failure to identify the culprit vessel, recanalization of the embolized bronchial artery, or progression of the underlying lung disease rather than the embolic agents used, which might explain why 500–750 μm microspheres displayed similar hemoptysis recurrence rates as 700–900 μm microspheres. Of note, we found that SPSs and the bronchiectasis subtype (the degree of cystic) were independently associated with recurrence. The possible explanations might include the following: (1) The use of small microspheres is potentially risky for nontargeted embolization of the viscera, so the use of particles smaller than 325 μm has been abandoned [21]. (2) Furthermore, with the help of using a microcatheter and placing it at the possible closest location near the abnormal vasculature, it decreased the possibility of nontarget embolization of the spinal artery as well as the risk of BAE-related neurologic complications. In addition, to complete the cessation of abnormal-looking bronchial arterial flow and stop bleeding, fewer bottles of large microspheres (700–900 μm) are used. Considering the comparative cost-efficacy, the use of 700–900 μm microspheres was associated with lower inpatient costs for patients undergoing BAE when compared with 500–750 μm microspheres, and it was more reasonable to recommend the use of 700–900 μm microspheres rather than 500–750 μm microspheres.

In terms of safety, there was no statistically significant difference in complications between BAE using 500–750 μm Embosphere microspheres and BAE using 700–900 μm microspheres, and no major complications were observed. The occurrence of minor complications, such as cough/expectoration, fever, chest pain, nausea/vomiting, allergy, ecchymosis at the puncture site, and dyspnea, was frequently observed in BAE procedures, consistent with findings from previous studies [4, 5, 8, 9]. One potential explanation for the absence of major complications is that the microspheres used in the procedure had diameters of either 500–750 μm or 700–900 μm, allowing them to be appropriately positioned in the pulmonary artery without causing occlusion of the normal peripheral branches. This feature reduces the likelihood of more severe complications (such as myocardial infarction or stroke) caused by ischemia.

Our study aimed to address a gap in the literature by conducting a comparative analysis of the efficacy and safety profiles of microspheres ranging from 500 to 750 μm and 700–900 μm, specifically in the context of bronchial artery embolization (BAE) treatment for hemoptysis patients. Our findings indicate that the 500–750 μm microspheres exhibit comparable efficacy and safety to the 700–900 μm microspheres in controlling hemoptysis. Additionally, considering the more favorable cost associated with the 700–900 μm microspheres compared to the 500–750 μm microspheres, our evidence suggests that the former may serve as a viable alternative in BAE treatment. However, the present study still has some limitations, including (1) considering the small sample size in our study, more patients from multiple centers are necessary for statistical validation; (2) a patient’s prognosis was also influenced by the management of their underlying respiratory disorders, such as antibiotic usage, blood oxygen levels, and inflammation index; (3) this is a retrospective observational study conducted in a single center. The choice of the diameter of the embolized microspheres is determined by the personal experience of different operators, which might result in a selective bias for embolic agents.

In summary, the utilization of BAE with 700–900 μm microspheres demonstrates similar levels of effectiveness and safety as BAE with 500–700 μm microspheres in the treatment of hemoptysis. However, it is important to note that the former option is less economically efficient. Consequently, it is advisable to prioritize the use of 700–900 μm microspheres for managing hemoptysis resulting from bronchiectasis.

Others♦ included IPA, ICA, IMA, TTA or CTA IPA inferior phrenic artery, ICA intercostal artery, IMA internal mammary artery, TTA thyrocervical trunk artery, CTA costocervical trunk artery, and SPSs systemic arterial-pulmonary circulation shunts. BA Bronchial artery.

### Electronic supplementary material

Below is the link to the electronic supplementary material.

Supplementary Material 1

## Data Availability

The data that support the findings of this study are available from the corresponding author upon reasonable request.
